# Transient behavior of compressed magnetorheological brake excited by step currents

**DOI:** 10.1038/s41598-021-91836-6

**Published:** 2021-06-09

**Authors:** Hongyun Wang, Cheng Bi, Yongju Zhang, Axiang Ji, Pengyuan Qiu

**Affiliations:** 1 College of Aeronautics, Taizhou University, Taizhou, 318000 Zhejiang China; 2 School of Mechanical and Energy Engineering, Zhejiang University of Science and Technology, Hangzhou, 310023 Zhejiang China

**Keywords:** Engineering, Materials science

## Abstract

Transient behavior of a magnetorheological brake excited by step currents under compression-shear mode has been experimentally studied. The results show that the amplitude of the applied current had little effect on the rising time of transient torque, while the rising time was significantly affected by the rotational speed, the compressive speed and the compressive strain position. The falling time of transient torque was independent of the amplitude of the applied current, the compressive speed and the compressive strain position, and it was affected by the rotational speed. The falling time of the transient torque was much shorter than the rising time by a step current. The transient process of MR brake applied as a step current was different from a stable process pre-applied at constant current in different particle chain structure forming processes. In addition, the compressive processes applied in one step current and randomly on/off current were compared and experimentally verified: the particle chains in two processes both experienced the same evolutionary of transient torque. The results achieved in this study should be properly considered in the design and control of magnetorheological brake under compression-shear mode.

## Introduction

Magnetorheological (MR) and electrorheological (ER) fluids are noted for the rapid transition, from liquid to solid-like material when an external field is applied^1–3^. The MR fluids can be used in a variety of industrial applications such as dampers, clutches/brakes, smart actuators and intelligent structures because of their controllable field-induced yield stress^4–6^. A MR brake/clutch may be a good replacement for the conventional disk brake/clutch. However, a traditional MR brake/clutch is designed a geometrical arrangement referred to as shear mode^7–12^. The magnitudes of the shear stress in systems with shear mode are too low to be of value to widespread commercial application of MR brake. The geometrical arrangement designated the squeeze mode has been found to produce compressive stress which are much higher than the yield stress in shear mode under the same magnetic field^13–18^, and this has generated new interests in this approach. Compressive behaviors of MR fluids have been investigated during recent years. Tao observed that a compression-aggregation process can modify the weak points at the ends of MR chains of MR microstructure under shear mode and enhance the yield strength of MR fluid^13^. Zhang et al*.* have studied the mechanism of squeeze-strengthen effect on MR fluids^14^. Mazlan et al*.* have experimentally investigated the influence of the magnetic field and gap size on MR fluid under compression mode^15,16^. Recently we have found that the compressive stress of MR fluids in compression mode was much higher than the shear yield stress in shear mode under the same magnetic field, and have compared the test results^17,18^.

Despite the fact that MR brakes have been investigated repeatedly under compression-shear, many of the studies dealt with the stable characteristics of MR brakes under constant currents^19–23^, as briefly noted here. Patil et al. have experimentally studied the influencing parameters of braking torque of MR brake in shear and squeeze mode^19^. Sarkar et al*.* have experimentally and theoretically studied the MR brakes under compression-shear mode^20,21^. The torque is found to be higher than only under shear mode and it is dependent on the pressure. Wang et al*.*^22^ and Wang et al*.*^23^ have studied the squeeze-strengthening effect of MR fluid in high-torque MR brakes. Their analyses show that the braking torques are tightly related to the squeezing stress, and the rotational speed is independent of the braking torque; A larger applied current, a higher squeezing stress will correspond to a higher braking torque.

MR fluids are notable for fast response time and continuously controlled rheological characteristics to applied currents. As widely recognized, a field-dependent yield stress of MR fluids is closely related to the structure of particles chains. When the shear stress exceeds the yield stress, it depends linearly on the shear rate^24^. Magnetostatic forces cause the particles to form chain and/or column structures along the direction of the applied field. The response time of structure formation of MR fluids is thought to be controlled by a competition between the viscoplastic behavior and the structure formation^25,26^. The dynamic characterization of ER fluids, analogous to that of MR fluids, has been theoretically and experimentally studied and found that the response time of shear stress is relate to shear rate, particle volume fraction, and applied electric field strength^27–34^. Tian et al*.* have investigated the dynamic responses of ER Fluid in shear mode and have found the rising time of the characteristic stress decreased with the increase of shear rate and particle volume fraction^27,28^. They also have experimentally studied the transient behavior of ER fluid under tensile and squeeze mode and have verified the effect of shear rate on the transient time^29,30^. Choi and Wereley have compared the characteristics of ER and MR fluid-based systems based on time response characteristics and dynamic range using nondimensional parameters^25^. Zhu et al*.* have proposed a Maxwell behavior model with the time constant to describe the normal force response of magnetorheological elastomer under compression mode, and have founded that the transient responses are affected by applied current, particle distribution and compressive strain^35^. Qi et al*.* have proposed a method to improve transient response of MR elastomer by incorporating carbonyl iron powder to possess Fe–Ni nano-flakes on its surface^36^. Wang et al*.* have proposed a novel parameter model to predicting the nonlinear hysteresis response of MR gel that is a new branch of MR materials^37,38^. Recently, we have found that the current has little effect on the rising time when we studied the performance of MR brake under squeeze-shear mode^23^. However, a thorough study of the transient response characteristics of MR brake under a step current or the current turned on/off randomly under compression-shear mode is still not complete. It is essential to clearly understand the transient response and parameter relationships of MR brake, which may provide a reference for the control of MR brakes and the design of new transmission systems under compression-shear mode. Our aims are to a better understanding of MR brake excited by a step current or the current turned on/off randomly under compression-shear mode, and to obtain the implications for the design and control of MR brakes involving frequently current on/off adjustment corresponding to different external conditions. In this research, the transient transmission behaviors of the MR brake under compression-shear mode excited by a stepwise current and the current turned on/off randomly were experimentally tested and discussed. They were also compared with those of stable behaviors. The structure strengthening effect was quantitatively discussed. An explanation based on the forming process of the chain structures was proposed to describe the time response characteristics of MR brake under compression-shear mode.

## Experimental setup

**Figure 1:**
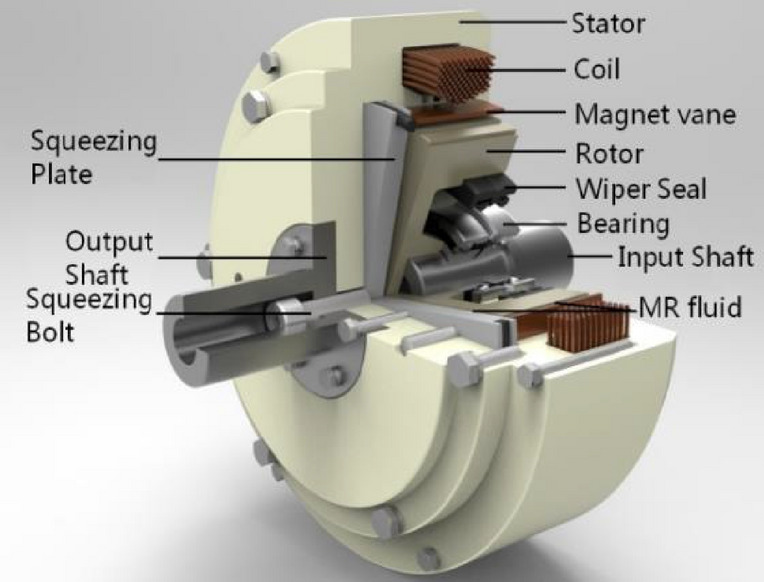
The schematic of the compression-shear type MR brake.

The test apparatus employed in the transient response of compression-shear type MR brake under step currents is similar to that employed in our former report^23^ as shown in Fig. 1. The squeezing plate has a diameter of 129 mm. A compressive force is produced by squeezing bolt. The MR fluid placed in the working gap *h*_1_ is squeezed along the magnetic field direction and then sheared, and that in the working gap *h*_2_, which is kept at a fixed distance of 1 mm, is always sheared. Main specifications of the proposed MR brake are given in Table 1.

**Table 1:** Specifications of the proposed MR brake.

Specification	Value
Type	Disk brake
Outer diameter	Dimethyl silicon oil
Thickness	3.09 (g/ml)
Major radius (*R*_*1*_)	65 mm
Minor radius (*R*_*1*_–*R*_2_)	20 mm
Major gap of MR fluid (*h*_1_)	1 mm
Minor gap of MR fluid (*h*)	1 mm
Coil turns	550 turns
MR fluid	MRF-2035

The braking torque (*T*) for proposed squeeze-shear mode of MR brakes is the sum of the torque induced in the gap of *h*_1_ and that in the gap of *h*_2_. Theoretical torque used for the same^23^ is

1 $$T = \frac{2\pi }{3}\left( {2R_{2}^{3} - R_{1}^{3} } \right)\tau_{0} \left( H \right) + \frac{{2\pi R_{2}^{3} }}{3}K_{H} P + \frac{{2\pi k\omega^{n} }}{n + 3}\left( {\frac{{R_{2}^{n + 3} }}{{h_{1}^{n} }} + \frac{{R_{2}^{n + 3} - R_{1}^{n + 3} }}{{h_{1}^{n} }}} \right)$$

where *τ*_0_ (*H*) is the yield stress of MR fluid; the radii of *R*_1_ and *R*_2_ are designed as 45 mm and 64.5 mm, respectively; *K*_*H*_ is a slope that increases with the field *H*; *P* is the compressive stress; the exponent *n* is called the shear thinning exponent; *k* is called the consistency; *ω* is angular velocity; And *h*_1_ and *h*_2_ are the initial gap sizes of MR fluid.

**Figure 2:**
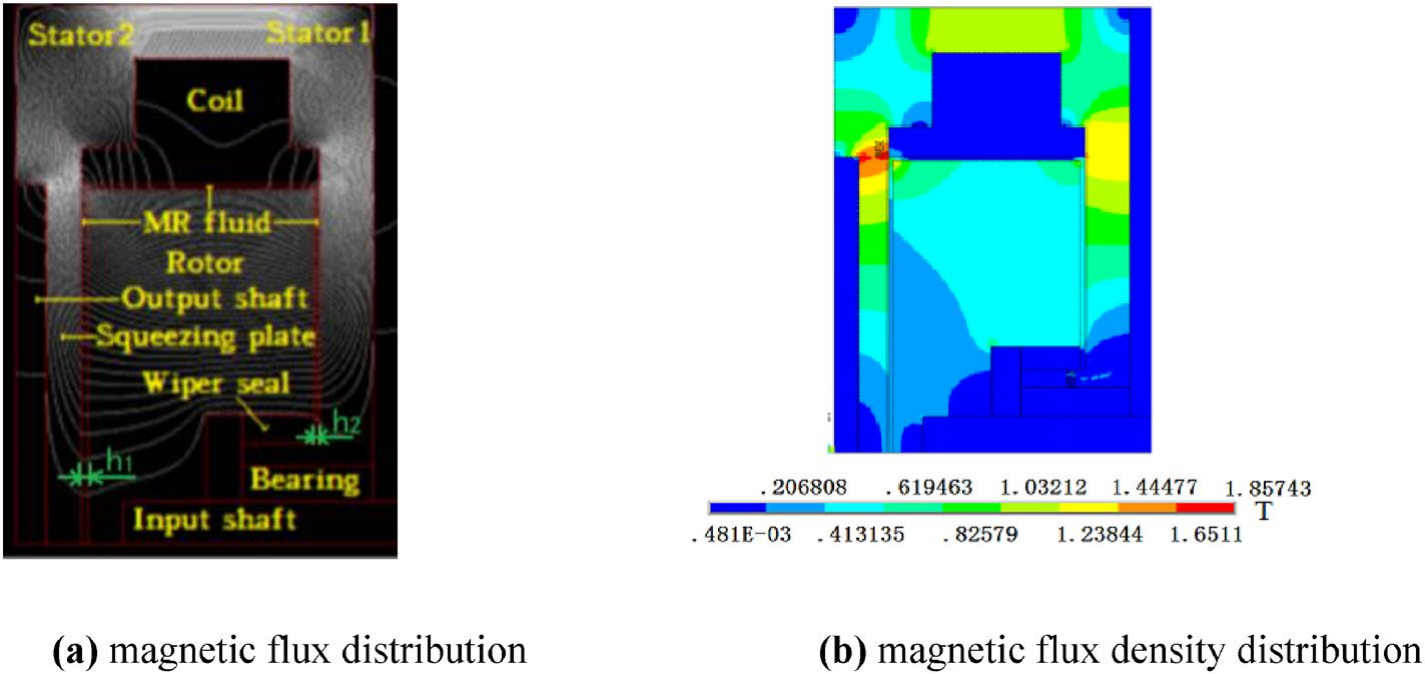
Results of the magnetic circuit simulations.

The results of the magnetic circuit simulations are shown in Fig. 2. The current is set as 1A. Combining with the material parameters of the MR fluid and the dimensions of the MR brake, the theoretical torque is calculated by Eq. (1) as 169 Nm at the initial gap size *h*_1_ and *h*_2_ of 1 mm, the compressive strain of 0.55 and the rotational speed of 90 rpm (*k* = 0.33 Pa s^n^, *n* = 0.885, *K*_*H*_ = 0.225^23^).

**Figure 3:**
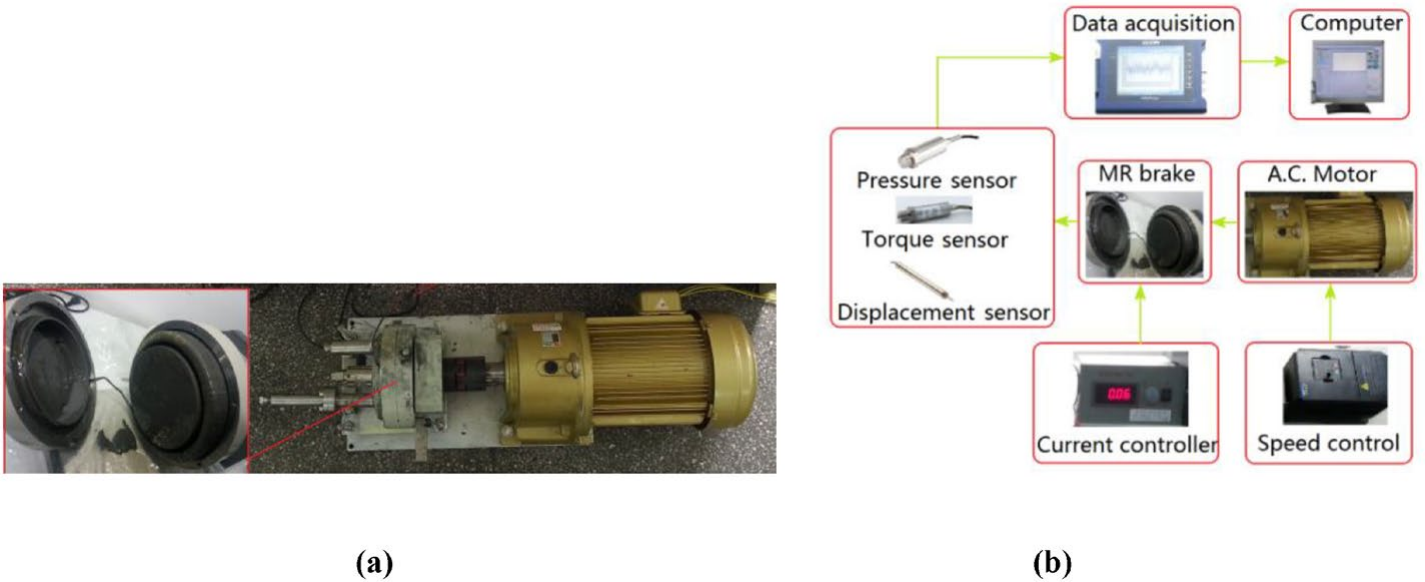
(**a**) An experiment prototype; (**b**) block diagram experiment layout of proposed MR brake.

Experimental setup is shown in Fig. 3a. The braking torque is measured by the dynamic torque sensor (TJN-4). The compressive stress acting on the MR fluid is detected by the pressure sensor (TJP-3). The compressive displacement acting on the squeezing plate is measured by the displacement sensor (500DC-SE, 0.25% FS). A DC power supply (PAB, range 0–36 V, 0–3 A) is used to switch on/off or adjust the current of the coil. An AC converter is used to control the rotational speed of motor. The compressive stress, torque and displacement values are gathered and processed by AVANT integrated data acquisition and analyzer (AVANT-MI-7008I), as shown in Fig. 3b. The accuracies of the torque sensor and the compressive stress sensor are 0.1% FS and 0.3% FS with the response time of 0.6 ms and 0.5 ms, respectively, which are much less than the response time of the MR brake, so it can be neglected.

To measure the transient response of the MR brake, firstly, a DC step current is suddenly applied on the MR brake before the moving of the squeezing plate, and then the MR fluid is sheared. At last, the current is turned off after the squeezing plate stops moving. The compression is carried out at an initial gap distance *h*_1_ = 1 mm and a compressive displacement is 0.6 mm. During the shear process, the rotational speed is kept constant.

Furthermore, experiments on an intermittently on/off applied current have been done. When a current of 1.0 A is suddenly turned on, compression and shear occur simultaneously. In this experiment, the compressive speed is 1.224 mm/s. The rotational speed is 90 rpm. The initial gap distance *h*_1_ is 1 mm. The compressive displacement is still 0.6 mm. Then the applied current effect on the transient process is determined. Four currents of 0.4, 0.6 0.8, and 1.0 A are employed. It is three processes that have been done: a pre-applied constant current, a step current, and random on/off currents. A MRF-2035 type of MR fluid from Ningbo Shangong Co. Ltd, China is employed in this investigation. It is based on iron powder and dimethyl silicon oil with a particle weight percentage of about 81%. All experiments are done at room temperature, 23 °C.

## Results

### Response time procedure

**Figure 4:**
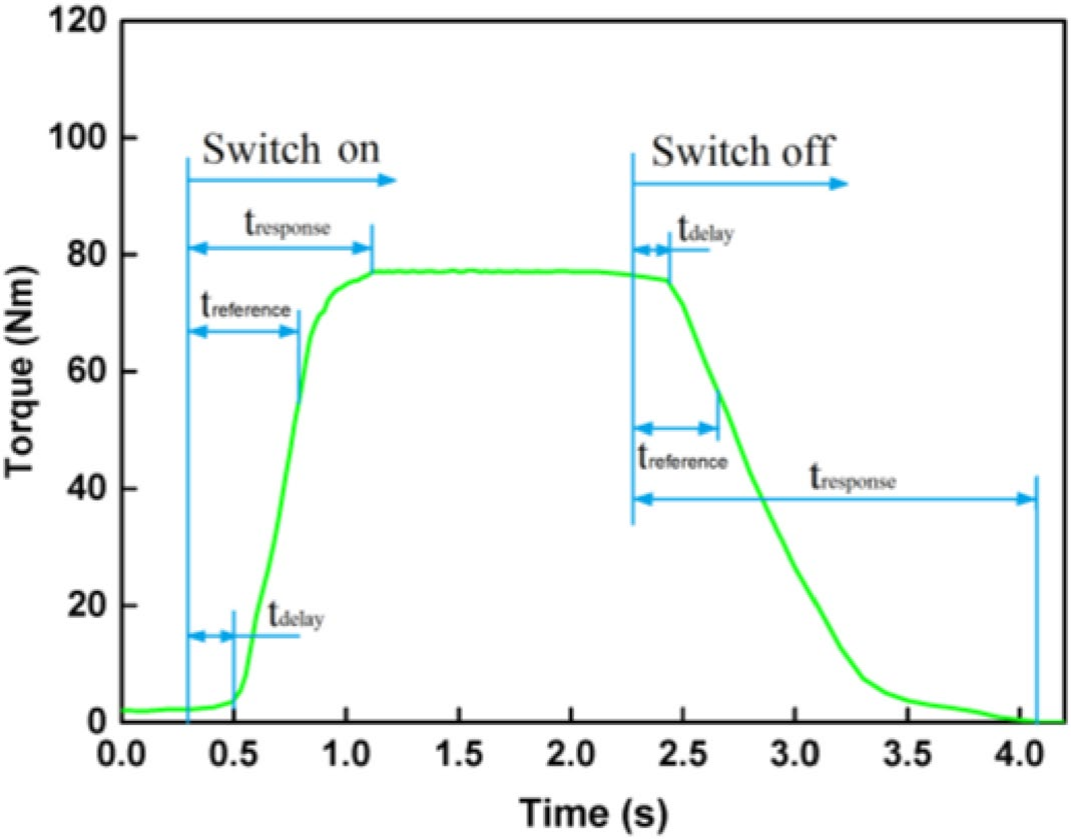
The typical definition of time parameters for the MR brake.

The response time of MR brake is not only determined by the properties of MR fluid itself and the driving electronics, but also is affected by other factors such as the working mode of the MR fluid (such as sheared, compressed), shear rate or velocity, temperature, and so on. Researchers use different time constants to define the transient performance for MR fluids or MR actuators^26,39,40^. Normally, the time to 63.2% of the stable value is defined as the time constant. As can be seen in Fig. 4, three time parameters *t*_*delay*_, *t*_*reference*_ and *t*_*response*_ are defined. *t*_*delay*_ is defined that the MR brake torque does not immediately change with the field, but is delayed by a time decay when an external magnetic field is imposed on or removed from the MR brake. *t*_*reference*_ is the time for the MR brake torque to acquire 1–1/e or 63.2% of its saturated value after the switching-on, and the time for the MR brake torque to decay to 1/e or 36.8% of its peak value after the switching-off, which is important parameter for designing and controlling of MR devices. *t*_*response*_ is the total response time from current turned on to the steady state. In our experiment, the time that the torque rises to 1–1/e or 63.2% or decreased to 1/e or 36.8% of the stable value is defined as the time constant.

### Response time affected by current

**Figure 5:**
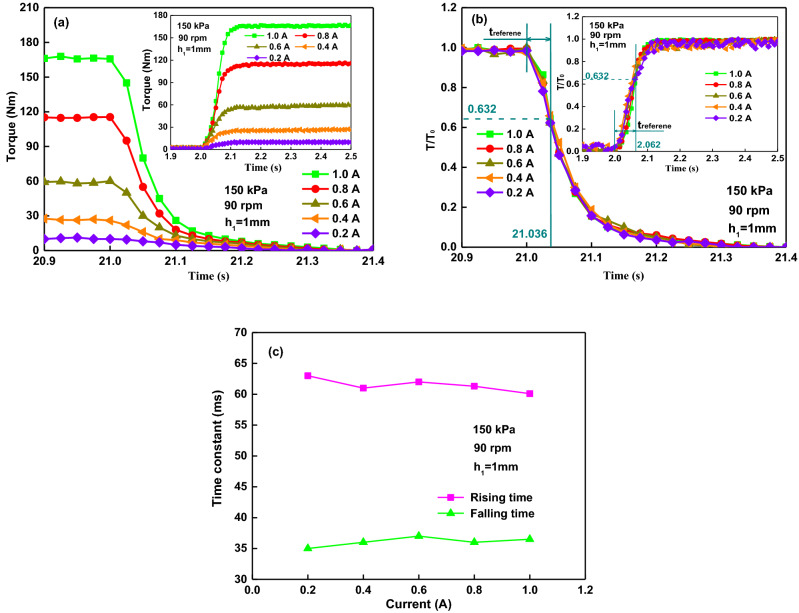
Torque response affected by current: (**a**) torque versus time; (**b**) normalized torque versus time; (**c**) rising and falling time constant versus the current.

Figure 5 illustrates the process of transient torque of MR brake under compression-shear at the applied currents of 0.2, 0.4, 0.6, 0.8 and 1.0 A with experimental parameters given above when the current is turned on at 2 s and turned off at 21 s. As shown in Fig. 5a, the torques decrease to zero in about 0.32 s. Inset: The torques rise rapidly at first and then slowly. To compare the characteristic response time of the torque under the different currents, the torque can be normalized as *T/T*_0_, where *T* is the instantaneous torque and *T*_0_ is the stable value. Figure 5b shows that the transient torque curves under different applied currents overlap each other well. It means that the response time of the torque is independent of the applied current. The torque rising times are about 62 ms (at 2.062 s) when the torques reach around 63.2% of the stable torque, and the falling time are about 36 ms (at 21.036 s) when the torques reach around 36.8% of the stable torque. The current effect on the time constant is shown in Fig. 5c. The results show that the two transient times of the currents have similar results. Different amplitudes of the applied current have little effect on the transient time of the rising or the falling. Moreover, the falling time constant (around 36 ms) is much shorter than the rising time constant (about 62 ms).

The response time of torque of the MR brake mainly includes the response of MR fluid, electromagnetic circuit and electro-magneto-mechanical coupling behavior of the MR brake. The response time of MR fluid is dependent on the concentration, the structure type of particles chains and distribution of magnetic particles, etc. It is very difficult or almost impossible to reduce the response time of MR fluid from the physical point of view. Due to the inhibitory effect to transient current of coil inductance, the current response time is dependent on the designing an electromagnetic circuit that includes the current drive and the coil configuration.

The electromagnetic coil in the MR brake is modeled by the inductance (482 mH) in series with the resistance (16.6 Ω) in the structure. For a step-up current at the rising time (*I* = 0 to *I* = *I*_*s*_) and at the falling time (*I* = *I*_*s*_ to *I* = 0), the time constants *λ* = *L/R* (the inductance *L* and the resistance *R*) at the rising and the falling process follow the single exponential function:

2 $$I_{r} \left( t \right) = I_{s} \left[ {1 - {\text{exp}}\left( { - \frac{t}{\lambda }} \right)} \right]$$

3 $$I_{f} \left( t \right) = I_{s} {\text{exp}}\left( { - \frac{t}{\lambda }} \right)$$

By applying the current from 0.2 to 1.0 A in steps of 0.2 A, the fitting parameters are given at the rising (R) and falling (F) process with Eqs. (2) and (3) in Table 2. With the increase of the applied current, the current response times are 11.63, 11.58, 11.57, 11.54, and 11.53 ms, respectively. The current fall times are 11.46, 11.43, 11.47, 11.41 and 11.45 ms, respectively. It means that the current rising and falling times have little difference with the increase of the applied current from 0.2 to 1.0 A because the current response time constant is in a time scale of milliseconds. The rising and falling times of torque have similar phenomenon with the increase of the applied current, as shown in Fig. 5c. By applying a step current, the performances of the MR fluid lead to the property changes of the MR brake. Therefore, the general trends of the torque response time of the MR brake are consistent with the MR fluid material and the electromagnetic circuit.

**Table 2:** Fitting parameters for the response characteristic according to Eqs. (2) and (3).

	0.2 A		0.4 A		0.6 A		0.8 A		1.0 A	
	R	F	R	F	R	F	R	F	R	F
Is (A)	0.19	0.21	0.39	0.41	0.61	0.62	0.78	0.81	0.99	1.12
Λ (ms)	11.63	11.46	11.58	11.43	11.57	11.47	11.54	11.41	11.53	11.45

### Response time affected by current rotational speed

**Figure 6:**
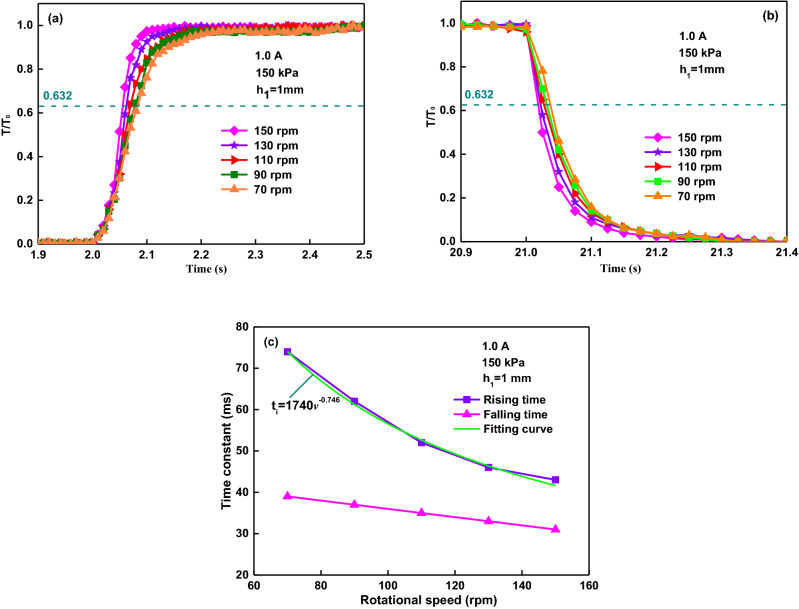
Torque response affected by rotational speed: (**a**) normalized torque versus rising time; (**b**) normalized torque versus falling time; (**c**) time constant versus rotational speed.

In the following, the rotational speed effecting on the time constant will be discussed. The normalized method to analyze the data by similar principles above will be applied. Figure 6a shows that the trend seems to be similar until the time reached 2.05 s. But afterward the normalized torque curves don’t overlap well each other. It means that a higher rotational speed corresponds to a smaller rising time constant. The faster the rotational speed, the easier the yield stress of MR fluid is to be reached, which will result in a quicker torque rising time. The falling time constants are apparently different when the different shear rates are applied, as shown in Fig. 6b. The larger shear rates mean to the smaller falling time constants. Also, when the shear rate increases, the response time decreases, as shown in Fig. 6c. The obtained rising and falling times are about 43–74 ms and 32–38 ms, respectively. It also shows that the rising time is much longer than the falling, and they both depend on the shear rate.

The shear rate dependence of response time to the shear rate is very significant for the response characteristic of MR brake. The response behavior of ER fluid depending on the shear rate can be used for reference^27–31^. It seems to have a similar influence on the MR effect. In the theoretical and experimental studies of the response behavior of ER fluid, the relationship between the torque response time and the shear rate has been achieved as^27,29^

4 $$t_{r} = {\rm K}\varepsilon^{ - 0.75}$$

where *t*_*r*_ is the torque response time constant, *K* is a parameter determined by the specific ER fluids, and *ε* is the shear rate. In our experiment of MR brake, the shear rate *ε* is calculated as

5 $$\varepsilon = 2\pi rn/6h$$

where *r* (65 mm) is the radius of the rotor, *n* is the rotational speed, *h* (1 mm) is the initial gap distance of fluid. At the compressive stress of 150 kPa and the applied current of 1.0 A, the changes of the time constants with shear rate can be depicted as shown in Fig. 6c. According to our experimental results, the fitting curve of rising time constant versus rotational speed is as

6 $$t_{r} = 1740n^{ - 0.75}$$

Equation (6) shows that the time constant is proportional to the rotational speed with an exponent of − 0.746 and it approximately satisfies the relationship of *t*_*r*_ ∝ *ε*^−0.75^. The higher the rotational speed, the shorter the transient time is, which agrees with the results for MR damper by Yao et al*.*^40^.

### Response time affected by compressive speeds

**Figure 7:**
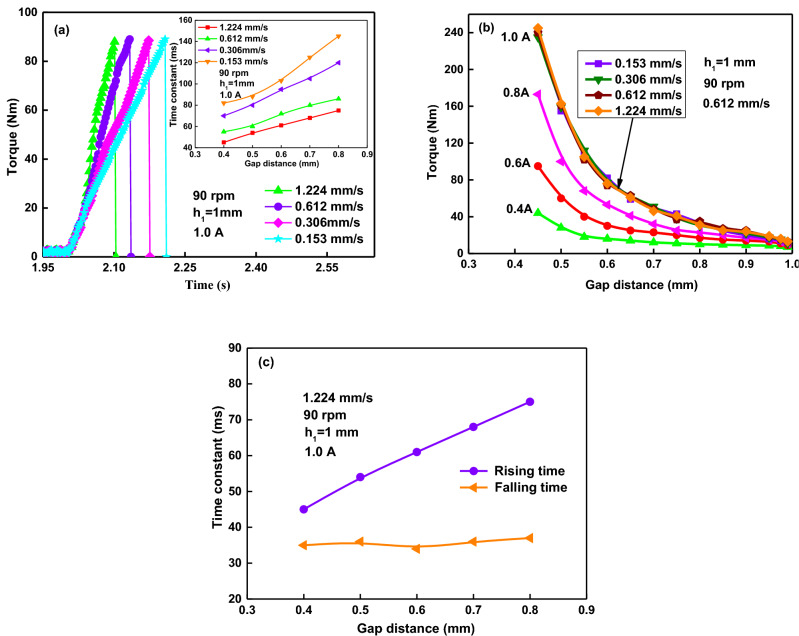
Compressive speed effect on transient rising torque process: (**a**) torque versus rising time; (**b**) torque versus gap distance; (**c**) time constant versus gap distance.

The tested curves of transient torque versus time with the compressive speeds of 1.224, 0.612, 0.306 and 0.153 mm/s are shown in Fig. 7a. It shows a higher compressive speed corresponding to a quicker torque rising time. It also indicates that the rising time constant decreases with the increasing compressive strain. That is to say, the rising time constant is shorter when the compressive speed is faster. The obtained rising time constant is about 45–76 ms, corresponding to the compressive strain of 0.6–0.2, at the compressive speed of 1.224 mm/s. The characteristic rising time constants of 88, 80, 61 and 54 ms correspond to the compressive speeds of 0.153, 0.306, 0.612 and 1.224 mm/s, respectively, at the compressive strain of 0.5. The curves of torque versus gap distance under the different compressive speeds and the different currents are shown in Fig. 7b. The curves overlap each other under the different compressive speeds, while the torques increase with the increasing applied current. It means that the compressive strain and the applied current are the critical factors on the magnitude of torque, while the compressive speed is a critical factor on the response time of the torque. The compressive speed has little effect on the falling response time, as shown in Fig. 7c. The falling time constants is around 35.6 ms. It is also much shorter than the rising time.

### Comparison between transient and stable compression-shear processes

**Figure 8:**
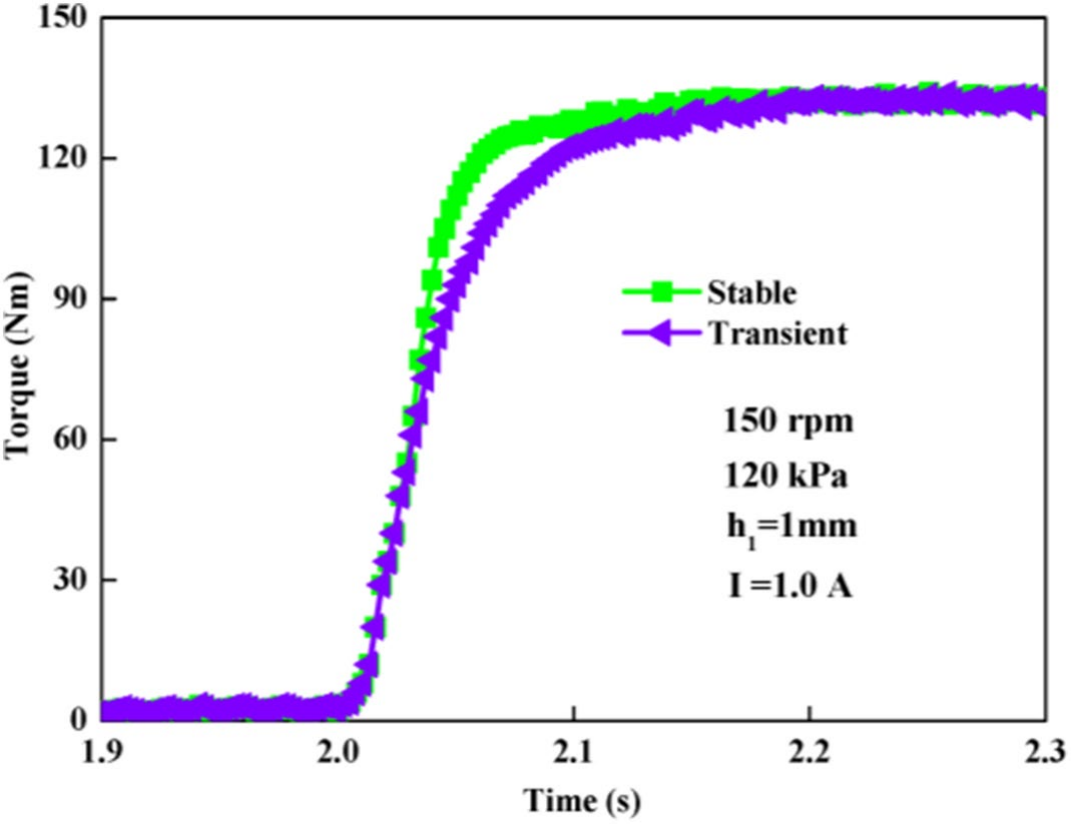
Comparison between transient and stable torque processes.

Namely, a stable compression-shear process is under a pre-applied current before a transient compression-shear begins, while a transient compression-shear corresponds to a suddenly applied step current. Recently, we have done experiments on compression-shear of MR brake at different initial gap distances^23^. The results show that a smaller gap distance results in a stronger structure strengthening effect of MR fluids and a higher torque. It is a stable and continuous compression-shear process with an initially applied current. In this transient investigation, the current is suddenly turned on and off under the initial gap distance of 1 mm. The typical results of a stable and a transient compression-shear process under the same conditions are shown in Fig. 8. At first the trends seem to be similar in 2.03 s, but afterwards the differences become bigger. The torques of the stable process in the beginning are higher than that in the transient process.

Table 3 compares the proposed brake in compression-shear and the brake^38^ in shear. The torque density is defined by the ratio of maximum braking torque to the overall dimensional volume of the brake. The designed brake provides a torque of 241 Nm, a torque density of 125.6 kN/m^2^ and a time constant of 58 ms. However, the brake in shear supplies a torque of 5.3 Nm, a torque density of 48.1 kN/m^2^ and a time constant of 50 ms. The torque of the proposed MR brake provides for a torque 45.5 times more and a torque density 2.6 times more compared to the brake in shear but the time constant is 1.16 times longer. It is promising for applications in many high-power situations.

**Table 3:** Specifications of the proposed MR brake and comparison to conventional MR brake.

Characteristic	Designed MR brake	Rossa et al*.*^11^
	In compression-shear	In shear
Radius (mm)	95.5	30
Length (mm)	67	39
Max. current (A)	1.0	0.9
Max.torque (Nm)	241	5.3
Torque/vol (kN/m^2^)	125.6	48.1
Time constant (ms)	58	50

**Figure 9:**
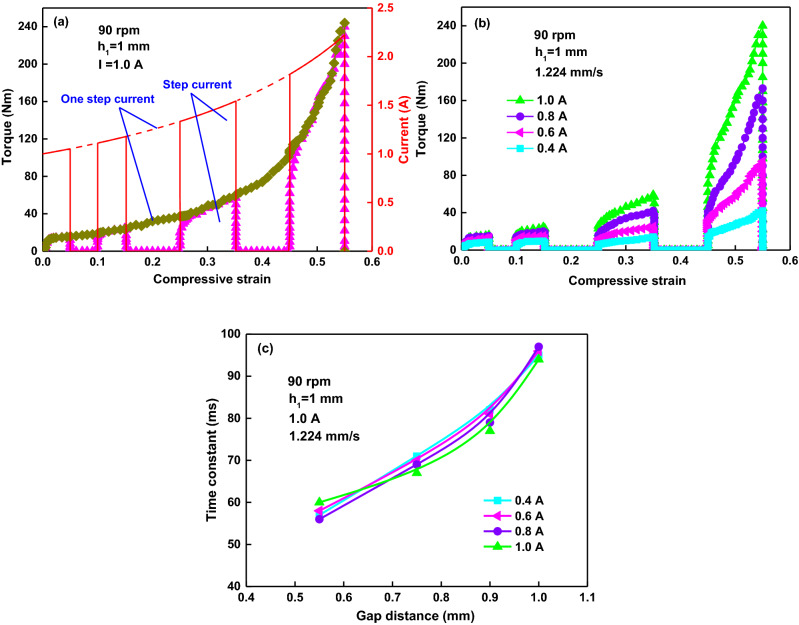
(**a**) Transient torque and instantaneous current versus compressive strain at a current of 1 A switched on/off between one and four times for the whole processes; (**b**) four test runs with four applied current that was switched on and off four times; (**c**) time constant versus gap distance under different applied currents.

Experiments on one step current and randomly on/off current with constant applied current of 1 A have also been done and been compared, as shown in Fig. 9a. It indicates that the amplitudes of torque under the current in two curves are close to each other and overlap corresponding to the same compressive strain. A typical results of intermittently turning currents on and off under different applied currents are shown in Fig. 9b. The curves after the first turning off of the current are still the transient compression. The rising time constants at applied currents of 0.4, 0.6, 0.8, and 1.0 A under the same gap distance of 0.75 mm are close to each other, around 69 ms, as shown in Fig. 9c. It indicates again that the amplitude of current still scarcely had any effect on the rising process of transient torque.

## Discussion

Tao experimentally studied the MR fluid microstructure by SEM images of iron-epoxy mixtures cured, and found that the MR fluid microstructure is dominated by single chains without the compression and it changes into thick columns after the compression^13^. The polarized particles form particle chains in the direction of the magnetic field, similar to that under shearing. Because the chains are not uniform and perfect (isolated chains, broken chains), the single chains are dominant in MR fluid’s microstructure. The weak points of MR microstructure are at the chain’s ends^13^. So the chains are more easily broken under shear. The particle chains of field-induced under compression get shorter and are pushed close to form a close-packed structure, and so the weak points of chains can be repaired under compression. The particle chains, which form the stronger and more robust BCT lattice structures, can bring a greater resistance and a much higher yield stress after compression when they are sheared. So, the total dynamic response of MR brake involves two distinct phases: formation and aggregation of the chain structures, at the moment of magnetic field-on; and another two distinct phases: depolarization and destruction of the chain structures, at the moment of magnetic field-off.

**Figure 10:**
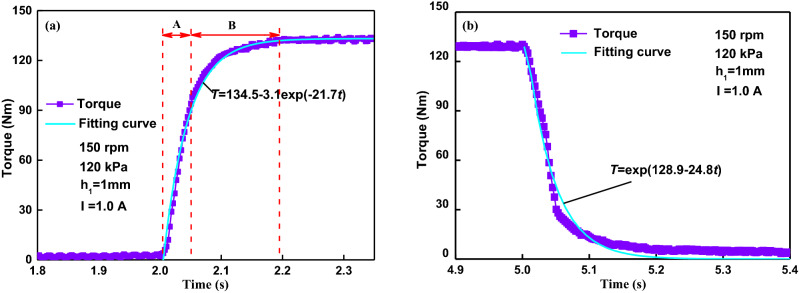
Transient torque versus time when the current is turned on at 2 s and turned off at 5 s. (**a**) Torque versus rising time; (**b**) torque versus falling time.

A complete transient torque response to a suddenly applied current turned on at 2 s and turned off at 5 s is shown in Fig. 10. The period of torque response can be divided into the two processes: A and B, as shown in Fig. 10a. The torque increases almost linearly with time in the initial period of *A* (about 49 ms) and then increases slowly in the period of *B* (about 141 ms), similar to the behavior of ER fluids in a transient shear flow^28^. The tested transient torque increases in a similar exponential shape with time and it can be well fitted by a exponential function of the time as *T* = exp(128.9 − 24.8*t*). Actually, it is more important that the changing chain structures of the characteristic rising time for the yield stress or the torque under a step current. The period of *A* can be ascribed to the chain forming process and that of *B* can be ascribed to the chain coarsening process^41,42^. When a magnetic field is applied, at first, the chain forming process of *A* from polarized particles is very fast. Then the aggregation process of *B* from single chains to aggregated chains and/or columns is slow in the presence of a shearing action, accompanying deformation and fracture of the aggregated chains and/or columns. This means that the aggregation time *B* of the chain structures is much longer than the forming time *A* of the chain structures. Thus, that induces the particle chains to form the thicker and stronger structures, which generates higher yield stress and leads to higher torque of MR brakes. So it is in this phase that the shear resistance of the MR brake increases.

In some industrial applications, the response of the brakes is highly demanded. The transient response is closely and directly related to the controllability of the torque of the MR brake. Therefore the ways to improve the response of the brakes can improve the performance of MR brakes. Table 4 shows the values of response time for different values of torque under different currents and rotational speed. These results are also presented in Figs. 5c and 6c. It shows that the torque increases faster when the higher current and rotational speed is applied. When a 0.2 A is applied on the MR brake, the torques of about 6.3 Nm is obtained, and the time constant is 75.4 and 44.3 ms at the rotational speeds of 70 and 150 rpm, respectively. When a 150 rpm is applied on the MR brake, the torques of about 6.3 and 104.3 Nm is obtained at the current of 0.2 and 1.0 A, and the time constant is about 44.3 and 43.1 ms, respectively. The torque is almost constant with the increasing rotational speed, and it increases with the increasing current. But the response time is very little affected with the increasing current, and it decreases with the increasing rotational speed. It also shows that the proposed MR brake has good performance in step response.

**Table 4:** Response time results for step control.

Current (A)	Rotational speed (rpm)	Torque (Nm)	Time constant (ms)
0.2	70	6.3	75.4
0.2	110	6.2	53.2
0.2	150	6.3	44.3
0.6	70	35.4	74.5
0.6	110	35.6	52.7
0.6	150	35.5	43.5
1.0	70	104.2	74.6
1.0	110	104.3	52.3
1.0	150	104.3	43.1

Figure 6a and b show that the curves overlap well each other in the time of 2.05 s, which mean that the forming process of chains is independent of the rotational speed. But afterward the curves, which don’t overlap, indicates that the chains coarsening is more affected by the rotational speed than the chain forming. Figure 7c shows that compression can bring a quicker response to external speeds, and the chain coarsening process is more affected by the shear rate while the chain forming process is determined by the compressive strain. The gap distance have great influence on the rising time constant because it forms the different length-diameter ratio of particle chain under the different gap distance. The small gap distance corresponds to the small length-diameter ratio of particle chain. This means the chain forming process in the smaller gap distance is much quicker than that in the larger gap distance. And the chain degeneration is largely determined and governed by the magnitude of the shear rate, rather than the compressive speed and the current. As shown in Fig. 10b, the torque decreases to almost zero in around 0.136 s when the current is turned off at 5 s, much shorter than the rising time. In Fig. 10b, the fitting curve of the torque as a exponential function of the time is *T* = exp(128.9 − 24.8*t*). Using the response time described above, the initial and final values of the torque are found to be about 1.29 Nm and 129 Nm respectively in the rising time at an activation current of 1.0 A and a compressive stress of 120 kPa. With the starting time of 2 s and 63.2% of the change in torque occurring at 2.045 s, the rising time is found to be 0.045 s. They are about 129 Nm and 5.43 Nm respectively in the falling time, and the falling time is found to be 0.032 s with the end time of 5 s and 36.8% of the peak value in torque occurring at 5.032 s. The falling time is much shorter than the rising, which means the depolarization and destruction of the chain structures was much quicker than the formation and aggregation of the chain structures during the loss of magnetic field.

In this study, the particle chains have been pre-formed and well structured at the beginning of the stable compression, so they can more easily form the more complete and robust structure during the compression-shear. For transient compression, there are no pre-structured particle chains. This is perhaps the most essential difference between them. So the stable torque is higher than the transient torque at the beginning. In Fig. 9a, the curves approximately overlapped each other during the compressive processes applied in one step current and randomly on/off current. This can be described that the particle chains in two processes both experienced the same evolutionary of transient torque. So the current switched on and off can not change the rapid formation of single chains followed by a slower coarsening of the chains, which leads to the similar amplitudes of torque.

## Conclusions

The transient behaviors of MR brakes under compression-shear have been experimentally studied. Effects on the transient torque process under different applied currents, different rotational speeds, different compressive speeds, and different compression strains have been determined. Upon applying a step current, the transient torque increased with time. Although the magnetic field strength greatly affected the amplitude of the torque, it showed little effect on the torque response time. A higher rotational speed and a larger compressive speed corresponded to a quicker rising response of MR brake. The amplitude of the compressive speed and compressive strain position had little effect on the falling time of the transient torque, while the falling time was greatly affected by the rotational speed. The transient torque of MR brake applied as a step current was also compared with the stable process pre-applied at constant current. The results show that the response time of MR brake was greatly affected by the method of applying the current, the compressive strain, and the working mode. These results are helpful for the design and dynamic control of MR brakes corresponding to the different external conditions.
